# TFF1 Induces Aggregation and Reduces Motility of *Helicobacter pylori*

**DOI:** 10.3390/ijms22041851

**Published:** 2021-02-12

**Authors:** Daniela Eletto, Megi Vllahu, Fatima Mentucci, Pasquale Del Gaudio, Antonello Petrella, Amalia Porta, Alessandra Tosco

**Affiliations:** 1 Department of Pharmacy, University of Salerno, 84084 Fisciano (SA), Italy; daeletto@unisa.it (D.E.); mvllahu@unisa.it (M.V.); fmentucci@unisa.it (F.M.); pdelgaudio@unisa.it (P.D.G.); apetrella@unisa.it (A.P.); 2 PhD Program in Drug Discovery and Development, University of Salerno, 84084 Fisciano (SA), Italy

**Keywords:** TFF1, *Helicobacter pylori*, motility, aggregates

## Abstract

Gastric cancer is considered one of the most common malignancies in humans and *Helicobacter pylori* infection is the major environmental risk factor of gastric cancer development. Given the high spread of this bacterium whose infection is mostly asymptomatic, *H. pylori* colonization persists for a long time, becoming chronic and predisposing to malignant transformation. The first defensive barrier from bacterial infection is constituted by the gastric mucosa that secretes several protective factors, among which is the trefoil factor 1 (TFF1), that, as mucin 5AC, binds the bacterium. Even if the protective role of TFF1 is well-documented, the molecular mechanisms that confer a beneficial function to the interaction among TFF1 and *H. pylori* remain still unclear. Here we analyze the effects of this interaction on *H. pylori* at morphological and molecular levels by means of microscopic observation, chemiotaxis and motility assays and real-time PCR analysis. Our results show that TFF1 favors aggregation of *H. pylori* and significantly slows down the motility of the bacterium across the mucus. Such aggregates significantly reduce both *flgE* and *flaB* gene transcription compared with bacteria not incubated with TFF1. Finally, our results suggest that the interaction between TFF1 and the bacterium may explain the frequent persistence of *H. pylori* in the human host without inducing disease.

## 1. Introduction

Gastric cancer represents one of the most common and deadly neoplasms in the world [1]. The evidence reports a strong association between gastric adenocarcinoma and *Helicobacter pylori* infection [2,3]. This Gram-negative bacterium normally colonizes the human gastric mucosa of 50–75% of the worldwide population, representing one of the most common pathogens [4]. However, only up to 10% of infected individuals develop duodenal ulceration and ultimately gastric cancer, whereas the majority remains asymptomatic [5,6]. The lack of symptoms does not alarm whom *H. pylori* infects and therefore in most cases there is a tendency to underestimate the presence of the bacterium. Its persistence in the stomach leads to a state termed “chronic superficial gastritis”, characterized by a profound inflammation that, along with other factors (age of infection, genetic traits, bacterial virulence and environmental factors), will then determine the outcome of the infection.

*H. pylori* is known to colonize specifically human gastric mucosa and particularly at the antrum and/or corpus level, depending on chemotaxis signals [7]. Its location is merely in the gastric pits and in the mucus layer rather than the epithelial cells and represents a unique feature that is receiving great attention. Indeed, the mucus plays a protective role against detrimental factors as low pH and pathogens act as primary immune defenses [8,9]. *H. pylori* co-localizes specifically with the mucin MUC5AC, secreted by normal gastric mucosal cells and thereby remains sequestered within the mucus layer [10,11]. Another factor that might participate in preventing *H. pylori* colonization is the trefoil factor 1 (TFF1).

TFF1 is one of the mucus components, known to interact with *H. pylori* and to play a protective role during infection. It is a member of the Trefoil Factor Family (the other two are TFF2 and TFF3) that is involved in mucosal protection by inducing repair and helping the formation of a stable mucus gel layer along with mucins [12,13]. It is constitutively secreted and co-expressed with the gastric mucin MUC5AC by mucus secretory cells of the gastric mucosa and is present in the stomach in three molecular forms—monomeric, homodimeric and heterodimeric (bound to a gastrokine 2 (GKN2), a IgG Fc binding protein (FCGBP) and an unknown protein/60 k) [14]. As the homodimer TFF1 binds to *H. pylori* [15] probably via its C-terminal region [16], which is also able to bind copper ions, this in turn promotes the formation of the homodimeric form [17]. *H. pylori* interacts with TFF1 through the lipopolysaccharides (LPS) in particular, the rough-form of LPS (RF-LPS), in a-pH dependent manner [18].

TFF1 is predominantly localized in the stomach, probably because it binds almost exclusively to gastric mucins and does not interact with colonic mucins [19]. Additionally, *Helicobacter* infection is characterized by host and tissue specificity, therefore their interaction was suggested to have a role in mediating the tropism of the bacterium for the stomach. Indeed, *H. pylori* also adheres to rat and bovine mucins, but natural infections in animals other than primates do not occur, probably because of physiological conditions, LPS composition of *H. pylori* and molecular features of TFF1 that determine such specificity of the bacterium. Given the high host specificity and tropism of *H. pylori*, it is still unclear if the interaction favors the bacterium persistence in the gastric environment or prevents the damage of the infection due to the protective functions of TFF1, or both.

Gastric biopsies from positive patients showed a reduced expression of TFF1 compared to uninfected ones, both at protein and mRNA levels [20,21]. However, these data are few and take into account only the chronic phase of infection. We recently demonstrated that TFF1 expression is differently regulated in a mouse model of *Helicobacter* infection. In particular, a downregulation is observed during the chronic phase, while an up-regulation of TFF1 characterizes the acute phase [22]. The over-expression of TFF1 during the acute phase is in agreement with cellular studies that report induction of TFF1 upon *Helicobacter* infection [23]. This observation, together with the protective effect of the gastrointestinal protein on the *Helicobacter*-induced damage [24,25], corroborates the beneficial role of the trefoil factor at the onset of infection.

Here we analyse, at a molecular and morphological level, the interaction between TFF1 and *H. pylori* in the context of mucus-secreting cells and we found a significant change in the motility of bacterium aggregates induced by TFF1.

## 2. Results

### 2.1. hTFF1 Binds H. pylori but Not H. felis

*H. pylori* is known to bind the homodimeric form of human TFF1 (hTFF1) and it has been suggested that the binding might contribute to the tropism of *H. pylori* for the stomach. Given the tissue- and species-specific *H. pylori* colonization, we hypothesized that the interaction of hTFF1–*Helicobacter* occurs preferentially with *H. pylori*. Therefore, we looked at the interaction of hTFF1 with bacteria by detecting the protein pulled down after bacteria sedimentation. We used two different *Helicobacter* strains (*H. pylori* and *H. felis*) and another Gram-negative bacterial strain (*E. coli JM109*). As a source of TFF1 protein we used supernatants from two mammalian cell lines (AGS-AC1, MCF7) and the human recombinant protein TFF1 (hrTFF1), produced and purified from the *E. coli BLR-DE3*-pLysS strain.

**Figure 1:**
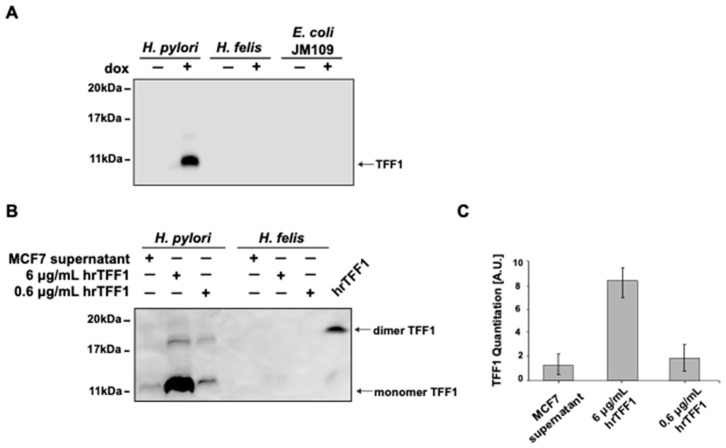
Trefoil factor 1 (TFF1) specifically interacts with *H. pylori*. Western blot analysis of pull-down experiments using (**A**) supernatants of AGS-AC1 clone induced or not with doxycycline, incubated with different Gram-negative bacteria; (**B**) TFF1 protein secreted by MCF7 cells or human recombinant protein TFF1(hrTFF1), incubated with *H. pylori* or *H. felis.* In the last lane hrTFF1 was loaded without reducing agents. (**C**) Densitometric analysis of *H. pylori* pull-down experiments in B. The experiments were repeated three independent times in technical duplicates.

As reported in Figure 1A, supernatants of inducible hyper-expressing AGS-AC1 clone, containing TFF1 upon doxycycline stimulation, show a specific interaction with *H. pylori*. Indeed, the only signal detected by the anti-TFF1 antibody via Western blot is observed when the supernatants were incubated with *H. pylori* and not with *H. felis* or *E. coli* (Figure 1A). A loading control of the experiment is reported in Figure S1. To corroborate these data, *H. pylori* and *H. felis* were incubated with two more sources of TFF1—supernatants from human breast cancer MCF7 cells, known to secrete high levels of TFF1, and the hrTFF1. The recombinant protein was used at two different concentrations, 0.6 and 6 µg/mL.

These concentrations were used because 6 µg/mL is more or less the supposed concentration present in the gastric mucus since its motogenic activity becomes measurable starting from this concentration [26]. Moreover, the protein contained in the MCF7 supernatant was estimated to be 3–6 µg/mL (Figure S2).

Our hrTFF1 protein is mainly dimeric (≈90%) as shown in the last lane of the Western blot of Figure 1B where it was analyzed in non-reducing conditions in order to preserve the disulfide bonds. While the protein secreted by MCF7 is mainly monomeric (>90%) [27]. To better compare results between recombinant protein and conditioned media we used also a ten-fold dilution, in order to realize a similar concentration of dimeric protein. As before, TFF1 was pulled down specifically by *H. pylori* and not by *H. felis* (Figure 1B). As expected, the signal intensity of MCF7 supernatants was comparable to 0.6 µg/mL hrTFF1 (Figure 1B), confirming that the dimeric form of the protein was pulled-down by the bacterium.

### 2.2. hrTFF1 Induces H. pylori Aggregation

**Figure 2:**
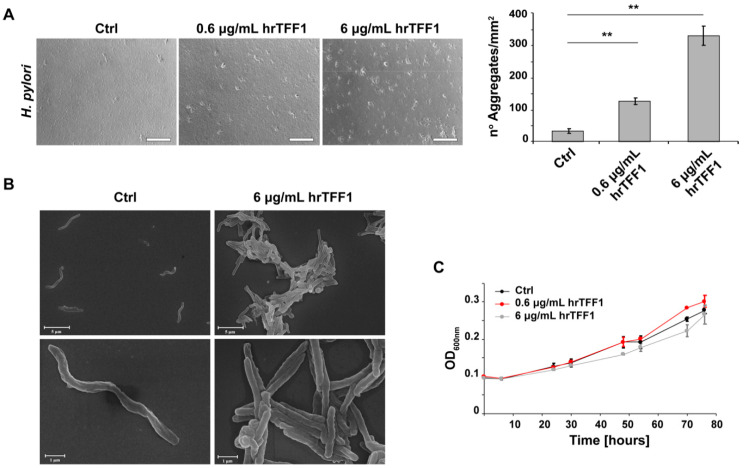
hrTFF1 induces formation of *H. pylori* aggregates. (**A**) Optical images of *H. pylori* incubated with or without 0.6 and 6 µg/mL of hrTFF1. Scale bar 150 µm. Panel on the right side reports the histograms quantification of bacterial aggregates. Data are mean of 3 independent experiments in technical duplicates (*n* = 6) ± SD. (*t*-test; ** *p* ≤ 0.01). (**B**) Scanning electron microscopy (SEM) images of *H. pylori* incubated with or without 6 µg/mL of hrTFF1. Scale bar—5 µm (upper panels), 1 µm (lower panels). (**C**) Growth curves of *H. pylori* incubated with or without 0.6 and 6 µg/mL of hrTFF1. Data are mean of 3 independent experiments in technical duplicates (*n* = 6) ± SD.

As TFF1 is up-regulated during early *H. pylori* infection [22] and can specifically bind it, we hypothesized that the interaction might serve to protect the gastric epithelium. To better understand the role of TFF1–*H. pylori* interaction, *H. pylori* cultures were incubated with two different concentrations of hrTFF1 (0.6 and 6 µg/mL) and the bacterial morphology was monitored over time by light microscopy. As shown in Figure 2A, *H. pylori* forms aggregates in a TFF1-concentration dependent manner within 4 h. Bacteria form large clumps only if incubated with the protein, whereas at the same time of observation without hrTFF1, bacteria were still predominantly in the planktonic form. We also corroborated this result recording the formation of aggregates over time (0, 30, 60, 120 min) and with multiple concentrations (0, 0.75, 1.5, 3, 6 µg/mL) of hrTFF1 (Figure S3). As expected, *H. felis* does not form hrTFF1-induced aggregates (Figure S4).

Bacterial aggregates were then observed by scanning electron microscopy (SEM) to examine *H. pylori* morphology. Figure 2B shows that the incubation of the bacteria with TFF1 is accompanied by a morphological transformation from a spiral-shaped to filamentous form, suggesting that the protein not only forces an interaction in the bacteria but also affects their morphology.

In order to explore if TFF1-dependent aggregation could influence *H. pylori* proliferation, we monitored growth of bacteria incubated with or without the protein. To avoid being misleading, the bacterial aggregates were fully dispersed by pipetting prior each optical density measurement. The co-incubation with the protein did not cause a statistically significant effect on the proliferation of *H. pylori* as the bacteria showed the same growth rate in any of the tested conditions (Figure 2C).

### 2.3. hrTFF1 Affects H. pylori Motility

**Figure 3:**
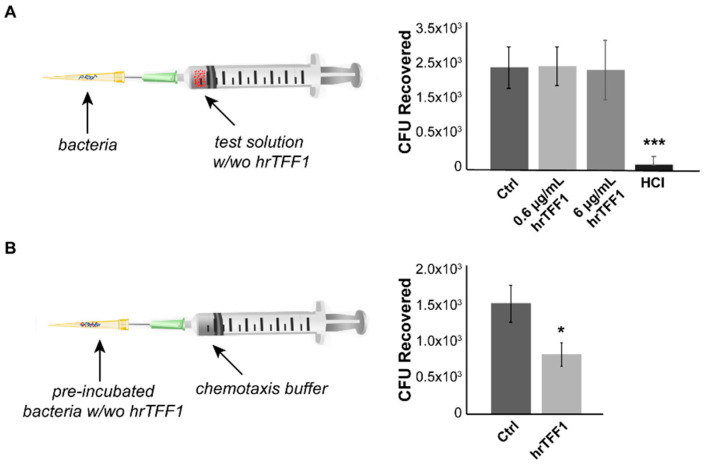
hrTFF1 affects *H. pylori* motility. (**A**) Left side, cartoon of the experimental procedure—bacterial suspension was put in the tip, while the syringe was filled with chemotaxis solution with or without 0.6 and 6 µg/mL hrTFF1. Right side, bacteria that have been moved from the tip towards solutions were recovered and counted as colony forming units (CFU). (**B**) Left side, cartoon of the experimental procedure—pre-incubated bacteria, with or without 6 µg/mL hrTFF1, were put in the tip, while the syringe was filled only with the chemotaxis buffer. Right side, bacteria that have been moved towards the syringe, were counted as CFU. Data are mean of 3 independent experiments in technical duplicates (*n* = 6) ± SD. (*t*-test, * *p* ≤ 0.05; *** *p* ≤ 0.001).

Once having assessed the formation of TFF1-induced aggregates, we hypothesized that this phenomenon could have an effect on the bacterium chemiotaxis and/or motility. To address this hypothesis, we first performed a capillary chemotaxis assay following the procedure reported by Cerda et al. [28]. The first question was whether TFF1 had chemoattractant or chemorepellent activity towards *H. pylori*. To this aim, the protein was loaded in the syringe and after 45 min of incubation all bacteria that moved from the disposable tip towards the syringe were recovered and plated on selective medium for bacterial colony counts. Figure 3A shows that TFF1 has no chemotactic effect on *H. pylori*, if compared to the buffer, while the positive control HCl works as a chemorepellent as expected.

The second question was whether TFF1-induced aggregation could influence *H. pylori* movements. To this aim, bacteria were pre-incubated with or without 6 µg/mL hrTFF1 for 1.5 h (the minimum time necessary to induce aggregation), placed in the disposable tip and attached to the syringe needle. As shown in Figure 3B, the number of bacteria recovered from the syringe in which *Helicobacter* was pre-incubated with hrTFF1 was significantly reduced compared to the control conditions (bacteria without hrTFF1), suggesting that TFF1, by inducing aggregation, reduces bacterial motility towards the chemotaxis buffer.

**Figure 4:**
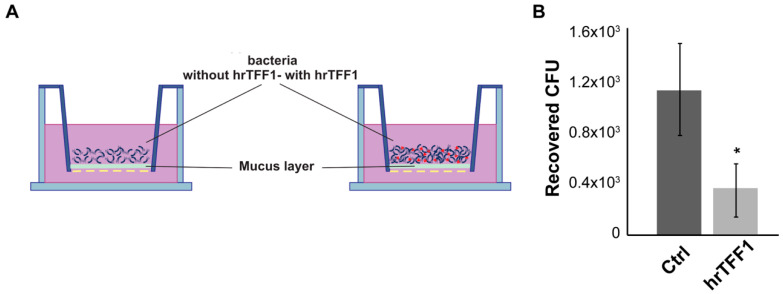
hrTFF1 delays motility of *H. pylori* across the mucus. (**A**) Graphical representation of the mucus motility assay. (**B**) Bacteria recovered from the lower chamber with or without pre-incubation with 6 µg/mL. Data are mean of 3 independent experiments in technical duplicates (*n* = 6) ± SD. (*t*-test, * *p* ≤ 0.05).

As the TFF1–*H. pylori* interaction occurs within the mucus, to further investigate the influence of the TFF1 protein on *H. pylori* motility, we set up a motility assay using the mucus collected from the HT29-E12 clone. The mucus was harvested after 21 days of culturing since it has been reported to contain high levels of TFFs and mucins [29]. Subsequently, it was layered on a 24 transwell filter insert and bacteria were set down on it (Figure 4A). Mucus per se slowed down the migration since the majority of bacteria took 30 min to reach the lower chamber, when directly layered on the transwell filter (data not shown). Figure 4B shows that pre-incubation with hrTFF1 protein significantly reduced the number of cells recovered in the lower chamber.

### 2.4. Transcriptional Regulation in hrTFF1-H. pylori Aggregates

**Figure 5:**
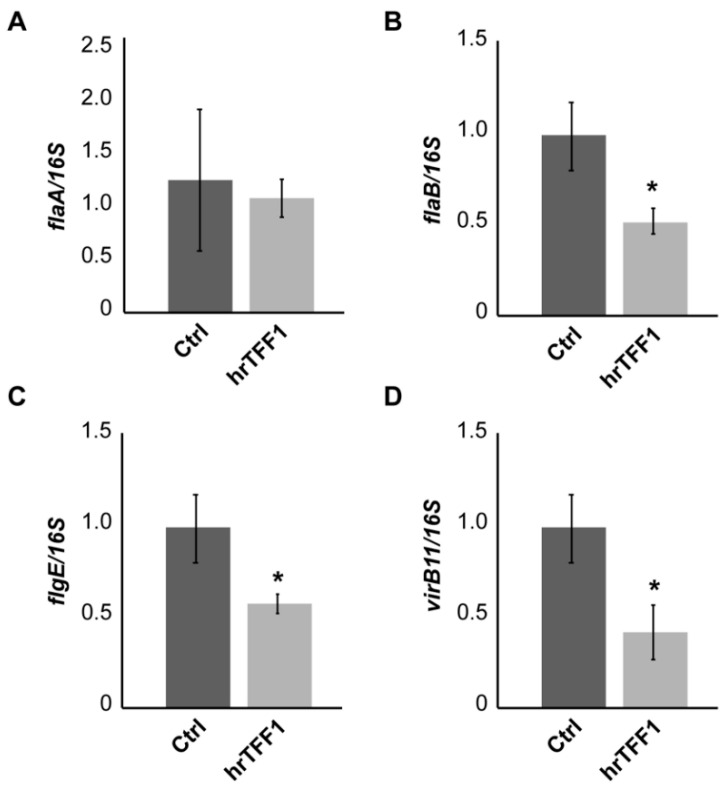
Real-time PCR analysis of *H. pylori* recovered from HT29-E12 infection. Panels (**A**)**–**(**D**) report expression level of *flaA*, *flaB*, *flgE* and *virB11*, respectively, in bacteria incubated with or without hrTFF1 prior infection. The 16S was used as a house-keeping gene. Data are mean of 3 independent experiments in technical duplicates (*n* = 6) ± SD. (*t*-test, * *p* ≤ 0.05).

Finally, we explored the possibility that the TFF1 interaction and the subsequent aggregation could affect *H. pylori* gene expression. To this aim, we used the aforementioned HT29-E12 clone infected for 24 h with *H. pylori*. Figure 5 shows that in bacteria incubated with hrTFF1 prior to infection, transcript levels of *flaB* and *flgE*, encoding components of the flagellum, were significantly reduced compared to the control. Similarly, *virB11*, encoding a component of the secretory apparatus complex virB/D, indispensable to translocate bacterial toxin to eukaryotic cells, was downregulated. However, the expression of other virulence genes (*alpA, alpB, hopZ, ureA, vacA, cag1, cagA, cag25*) did not change compared to the control (data not shown).

The reduced expression of the flagellum components is consistent with the compromised motility of *H. pylori* TFF1-dependent aggregates.

## 3. Discussion

The ability of *Helicobacter pylori* to bind TFF1 was previously demonstrated; different strains of *H. pylori* were found to interact with the dimeric form of the protein, while other Gram-negative bacteria did not [15]. We corroborated the existence of this interaction, also demonstrating that *H. felis*, another species of the *Helicobacteraceae* family, was not able to bind the TFF1 protein. This additional result is in line with the hypothesis that *H. pylori* co-evolved with its host for at least 100,000 years [30], and suggests that the interaction between TFF1 and *H. pylori* could have a role not only mediating the tropism of the bacterium within the stomach [31], but also affecting the fitness of *H. pylori* in different human hosts and explaining the frequent asymptomatic persistence.

*H. pylori* interacts with the dimeric form of TFF1 through its lipopolysaccharide [18]. The LPS is typically composed of three domains—the hydrophobic lipid A (or endotoxin), which anchors the molecule in the outer membrane; the core-oligosaccharide (which has been divided into the inner and outer core) and the variable O-antigen extending from the cell to the external environment.

The HP1191 mutant, lacking the heptosyltransferases responsible for the addition of the second LD-Hep residue of the core oligosaccharide, is no more able to bind TFF1 [29], moreover the purified Rough Form (RF) of LPS (without the O-antigen) and not the Smooth Form (SF) was able to bind the protein [18].

The core oligosaccharide region of *H. pylori* LPS has been recently redefined, suggesting that it comprises only the inner core conserved hexasaccharide of the previous model, while the O-antigen domain includes the outer core structure of the previous model [32], and according to Li et al. the conserved motif in the core oligosaccharide is essential for colonization. Considering these new findings, it seems necessary to better define the structure of the RF-LPS able to bind TFF1.

Although *H. pylori* and *H. felis* share the same sugars in the core oligosaccharide, they have different monosaccharide abundance [33]. Further investigations are required in order to better characterize the exact sugar or sequence of sugars that directly interacts with TFF1. However, our results suggest that LPS species-specific structure may be indicative of adaptive mechanisms that allow the bacteria to persist in their specific host.

To further investigate the effect of TFF1 binding on the bacterium, we first observed bacteria by light microscopy and highlighted that the formation of aggregates is incubation time- and TFF1 concentration-dependent. It has also been reported that human gastric mucins [34], and Galectin-2 [35] are able to induce aggregation, the former limiting bacterial proliferation and the latter exerting a bactericidal activity. Hence, we followed bacterial growth in the presence of TFF1, but it did not show any bactericidal activity nor inhibition of proliferation. Indeed, when we observed *H. pylori* morphology by means of scanning electron microscopy, we found that the bacterium does not keeps its spiral shape, but the clump formation is accompanied by a morphological transition into filamentous forms. To our knowledge, the function of elongated *H. pylori* cells has not been determined. However, as it has been suggested for the formation of aggregates/biofilms, this morphological transformation may play a protective role against adverse environmental factors. In particular, it has been suggested that the formation of aggregates could also be beneficial for bacteria, protecting them from antibiotics and also favoring the development of resistance, and for this reason disruption of aggregates during the eradication therapy could make it more effective [34].

Interesting, the aggregate formation was associated with the dysregulation of *virB11*, a virulence gene encoding a component of the secretory apparatus complex, virB/D, while, the expression of other virulence genes required for adhesion and invasion, such as *alpA, alpB, hopZ, urea, vacA, cag1, cagA and cag25*, did not change.

Furthermore, following the hypothesis that TFF1 could affect bacterial movements, we investigated the potential chemotactic effect of the protein on the bacteria but we did not observe any modification of bacterial migration towards the protein, compared to the control buffer. On the other hand, when bacteria were pre-incubated with TFF1, their migration towards the buffer was significantly impaired. To better corroborate this evidence, we set up a motility assay measuring the bacteria motility through a mucus layer, mimicking a physiological environment. Mucus represents a natural protection that strongly prevents bacterial movements. Mucus samples isolated from healthy and diseased individuals have shown differences in micro-rheological properties, as in patient with tumors the mucus appears less viscous and more permeable to bacteria that can swim faster [36]. For our purposes, we used mucus produced by the HT29-E12 clone, which is not exactly as the native one, but contains MUC5AC, TFF1 and TFF3 [29]. We found that the mucus by itself impaired bacteria movements and pre-incubation of *H. pylori* with TFF1 significantly slowed down its migration throughout the mucus.

*H. pylori* is a lophotrichous bacterium with 4-8 flagella. Helical shape and flagellar-based motility are used by *H. pylori* to penetrate the viscous mucus layer and colonize the stomach [37].

The bacterial flagellum is composed of multiple types of protein and among these FlgE is the main protein of the flagellar hook, while FlaA and FlaB are the components of the flagellar filament. Since strains lacking the *flgE* gene and *flaA* or *flaB* genes showed no motility or exhibited lower motility, respectively [38,39], we investigated whether TFF1 could affect their expression. As expected, in TFF1-dependent *H. pylori* aggregates, both *flgE* and *flaB* genes were significantly less transcribed compared to bacteria not incubated with TFF1, while *flaA* gene transcription appeared to be not affected. This is not surprising since *H. pylori* flagellar-related genes are divided in three classes and their transcription is controlled by three different RNA polymerase sigma factors—σ80 (RpoD), σ54 (RpoN) and σ28 (FliA). In particular, the two flagellins FlaA (the major constituent) and FlaB (the minor constituent) are regulated by σ28 and σ54 factors, respectively. They are both necessary for full motility [39], but they can be differently regulated by environmental factors.

In conclusion, although most virulence factors were not affected, the ability of TFF1 to induce the formation of aggregates in which bacteria lose their spiral shape and exhibit reduced motility may explain the frequent persistence of *H. pylori* in the human host without inducing disease.

## 4. Materials and Methods

### 4.1. Cell Cultures

HT29-E12, a mucus secreting sub-clone of the human colorectal adenocarcinoma cell line HT29-MTX (a generous gift from Professor Per Artursson, Uppsala University, Uppsala, Sweden), AGS-AC1, a TFF1 inducible hyper-expressing clone previously described [40] and MCF7 (breast cancer cell line).

The aforementioned cell lines were cultured in Dulbecco’s Modified Eagle Medium (DMEM, Euroclone, Austria), supplemented with 10% (*v*/*v*) fetal bovine serum (FBS, Euroclone, South America origin, EU approved), 100 U/mL penicillin and 100 μg/mL streptomycin (Euroclone, France). Medium for AGS-AC1 was supplemented with 600 μg/mL G-418 (Sigma-Aldrich, Saint Louis, MO, USA) and TFF1 expression was induced with 1 μg/mL of Doxycycline (Sigma-Aldrich, Saint Louis, MO, USA). All cell lines were maintained at 37 °C in a 5% CO_2_ atmosphere.

### 4.2. Bacterial Strains and Culture Conditions

*H. pylori* P12 strain, kindly provided by Dr. Marguerite Clyne (University College Dublin), and *H. felis* strain ATCC 49179, were cultured on selective Columbia agar (Oxoid, Basingstoke, Hampshire, UK) containing 7% (*v*/*v*) defibrinated horse blood (Oxoid, Basingstoke, Hampshire, UK) supplemented with antibiotic mix (DENT or Skirrow, respectively, Oxoid, Basingstoke, Hampshire, UK). Bacteria plates were incubated for 3–4 days in a capnophilic atmosphere with 10% CO_2_. Once grown on the plate, bacteria were scraped using brain heart infusion (BHI Oxoid, Basingstoke, Hampshire, UK) and measured at optical density at 600 nm (OD_600_) considering 1 OD_600_ = 1 × 10^8^ bacteria/mL.

*Escherichia coli* strains (JM109 and DH5α) were grown in LB (10 g/L tryptone, 5 g/L yeast extract and 5 g/L NaCl, pH 7) under shaking at 37 °C.

### 4.3. Human Recombinant TFF1 Production and Purification

*E. coli* BLR (DE3) pLysS containing the hrTFF1-pIVEX vector, encoding the human recombinant TFF1, were inoculated in LB liquid medium supplemented with 100 μg/mL of Ampicillin (Amp) and incubated for 16 h at 37 °C under shaking. After 24 h, 20 mL of bacterial culture was diluted in 1 L of LB liquid medium supplemented with 100 μg/mL of Amp and incubated at 37 °C under shaking. When the culture reached an OD_600_ of ≈0.8, protein expression was induced by adding 1 mM Isopropyl β- d-1-thiogalactopyranoside (IPTG), and growth was allowed for 2 more hours. After IPTG-induction, bacteria were harvested by centrifugation at 4000 *g* for 20 min at 4 °C and washed twice with 30 mL of PBS.

Briefly, bacteria were resuspended in buffer A (20 mM sodium phosphate buffer, pH 7.4, 500 mM NaCl, and 30 mM imidazole) containing a protease inhibitor and sonicated for 40 min (1 min on/1 min off, at 24% of amplitude) on ice. The lysate was clarified by centrifugation at 10000 *g* for 30 min at 4 °C, and filtered through a 0.45 μm filter (Millipore, Bedford, MA, USA) and the his-tagged hrTFF1 protein was initially purified by affinity chromatography on 1-mL HisTrap HP column (Amersham Biosciences, Uppsala, Sweden) using an AKTA Purifier chromatographic system (Amersham Biosciences, Uppsala, Sweden). The eluted fractions corresponding to the chromatographic peaks were analyzed by SDS-PAGE on 18% acrylamide gel, stained with Coomassie blue and those containing the his-tagged protein were pooled and subsequently digested with the Factor Xa (Promega, Madison, WI, USA) for 30 h at 25 °C to remove the his-tag. After digestion the protein was loaded again on the His-trap HP column to definitely separate it from the cleaved tag.

### 4.4. Pull-down Interaction Experiment

Cell supernatants from MCF7 or AGS-AC1 or human recombinant TFF1 (hrTFF1) (0.6 and 6 μg/mL) were incubated with bacteria suspension (5 × 10^7^/mL) of *H. pylori*, *H. felis*, *E. coli* JM109 or DH5α in 6-well plate for 4 h at 37 °C. At the end of incubation, bacteria were collected, centrifuged at 4000 *g* for 5 min, washed with PBS (Euroclone) and centrifuged again. The pellet was resuspended in 50 µL of Laemmli buffer, sonicated and centrifuged at 10000 *g* for 10 min at 4 °C to remove cellular debris. Then, 5 μL of each lysate was analysed by Western blot and TFF1 probed by polyclonal anti-TFF1 antibody [17]. Immunoblots were developed by chemiluminescent reaction and detected by LAS 4000 (GE Healthcare Life Sciences, Waukesha, WI, USA) digital imaging system. Immunoblot bands were quantified by ImageQuant TL software version 1.2 (GE Healthcare Life Sciences, Waukesha, WI, USA). Each experiment was repeated three independent times in technical duplicates.

### 4.5. Helicobacter pylori Growth Curve

*H. pylori* suspension of 1.25 × 10^7^/mL was incubated with hrTFF1 (0.6 and 6 μg/mL) or with an equal volume or vehicle (PBS), in a 24-well plate. At 6, 24, 30, 48, 54, 70 and 76 h bacterial growth was monitored by measuring optical density at 600 nm (OD_600_) (Multiskan spectrum, Thermo, Vantaa, Finland). The experiment was repeated three independent times in technical duplicates. The results are expressed as means ± SD. Statistical differences between the groups were evaluated by two-way ANOVA. Statistical analyses were done using PRISM4 software (GraphPad Software, La Jolla, CA, USA). A *p* < 0.05 was considered statistically significant.

### 4.6. Bacterial Aggregation Observation

*H. pylori* (7.5 × 10^6^/mL) was incubated with or without hrTFF1 (0.6 and 6 μg/mL) for 6 h in a 12-well plate. The experiment was repeated three independent times in technical duplicates. At 6 h post-incubation, six pictures per well were taken by AME-3206 Digital inverted Microscope (AMG/EVOS, Mill Creek, WA, USA), at 20× magnification. *H. pylori* aggregates were quantified by ImageJ 1.49 software by using the following method—convert to 8-bit image, apply a moments threshold (0 to 100 boundaries), and analyze clumps with a size between 100 to infinity and a circularity between 0.10–1.00.

### 4.7. Scanning Electron Microscopy Analysis

*H. pylori* was grown as described above, collected from the plate and incubated in BHI broth supplemented with 10% FBS, at 0.1 OD_600_. After 24 h, 1 × 10^6^ bacteria were put on coverslips pre-treated with 0.01% (*v*/*v*) poly-l-lysine (Sigma–Aldrich, Saint Louis, MO, USA) in 24-well plates and incubated with or without hrTFF1 6 μg/mL. After 16 h, medium was removed and bacteria fixed by 25% glutaraldehyde (*v*/*v*) for 16 h at room temperature. Samples were then washed and dehydrated in graded alcohol of 25%, 50%, 75%, 95% for 5 min and three times with 100% ethanol for 10 min. Finally, each coverslip was mounted on the SEM stage and analyzed. Samples were sputtered with gold using a LEICA EMSCD005 metallizator producing a deposition of a 100–200 Å thick gold layer. SEM images of each sample were then acquired using a Tescan Solaris UHR microscope equipped with secondary electron and backscattered electron detectors (TESCAN, Brno, Czech Republic). Analyses were conducted at 5 keV.

### 4.8. Chemotaxis Assay

#### 4.8.1. Analysis of TFF1 Chemiotactic Properties

Chemotaxis assays were carried as described by Mazumder [41] and revised by Cerda et al. [28]. Bacteria were grown as described above, collected from the plate and inoculated in BHI broth supplemented with 10% FBS, starting from 0.8 OD_600_. After 24 h, bacteria were diluted in chemotaxis buffer (10 mM potassium phosphate, pH 7.0; 3.0% polyvinylpyrrolidone) up to a concentration of 4 × 10^7^ bacteria/mL (0.4 OD_600_) and 100 μL of bacterial suspension was drawn into a disposable 200 μL pipette tip. A 100 μL volume of solution to be tested (10 mM HCl, hrTFF1 6 μg/mL, hrTFF1 0.6 μg/mL diluted in chemotaxis buffer) was drawn up into a 1-mL tuberculin syringe and a 23 G × 1¼ stainless-steel needle (0.6 × 30 mm) was used as the chemotaxis capillary. Chemotaxis buffer alone was included as control. The needle-syringe system was positioned into the pipette tip and incubated horizontally at 37 °C under microaerophilic conditions for 45 min. At the end of incubation, bacteria recovered from the syringe were appropriately diluted and plated onto Columbia Blood Agar (Oxoid, Basingstoke, Hampshire, UK) plates supplemented with 10% FBS (Euroclone) and DENT (Oxoid, Basingstoke, Hampshire, UK). Colonies were counted after 4–5 days of incubation in capnophilic atmosphere with 10% CO_2_. The experiment was repeated three independent times in technical duplicates.

#### 4.8.2. Analysis of TFF1 Influence on H. pylori Chemotactic Behavior

The experiment was similar to the above described except for starting bacteria that were diluted up to a concentration of 1 × 10^7^ bacteria/mL (OD_600_ = 0.1) and preincubated for 1.5 h *w*/*w* hrTFF1 (6 μg/mL), in order to favor the formation of aggregates.

Proper dilutions of the syringe content were plated for CFU (colony forming unit) counting. The experiment was repeated three independent times in technical duplicates.

### 4.9. Motility Assay

HT29-E12, mucus-secreting cells, were used as mucus producers. Once they reached confluence, day 1 started and after 20 days of culturing, replacing the medium every two days, antibiotic-containing medium was replaced with antibiotic-free medium and the mucus was collected every 2 days, avoiding mechanical stress. Once we obtained a sufficient quantity of mucus, 100 μL of slurry (1:1, mucus: BHI broth) was carefully set down on the filter of a transwell system (Falcon^®^ 353097; pore size 8 μm, Merck, Millipore). The lower chamber of the transwell was filled with 500 μL of BHI supplemented with 10% FBS.

Bacteria were grown as described above collected from the plate and grown in BHI liquid broth supplemented with 10% FBS, starting from 0.1 OD. After 24 h, 100 μL of 1 × 10^6^ bacteria/mL (1 × 10^5^ bacteria/well) was preincubated for 1.5 h with or without hrTFF1 (6 μg/mL), in order to favor the formation of aggregates and then layered on the previously prepared mucus. The system was incubated at 37 °C under microaerophilic conditions and after 24 h 50 μL of the lower chamber medium was plated for CFU counting. The experiment was repeated three independent times in technical duplicates.

### 4.10. Real Time-PCR

HT29-E12 cells were cultured for 20 days before enrolling in this experiment. A total of 24 h prior to infection, antibiotic-containing medium was replaced with antibiotic-free medium and cells were infected with *H. pylori* with MOI 1:30 (7.5 × 10^8^ bacteria/well) in a final volume of 500 μL RPMI +10% FBS without antibiotics. Before adding bacteria to the cells, *H. pylori* was pre-incubated without or with 6 μg/mL hTFF1 for about 2 h. Bacterial RNA was extracted using TriPure Isolation Reagent (Sigma-Aldrich), reverse transcribed (500 ng) into cDNA with M-MLV Reverse Transcriptase (GeneSpin S.r.l, Milan, Italy). The Real-Time PCR was performed using the Light Cycler 480 II instrument (Roche, Basel, Switzerland). Suitable dilutions of cDNA were used for each gene in a 12 μL reaction using StoS Quantitative Master Mix 2X SYBR Green (GeneSping S.r.l). Primer sequences are reported in Table S1. Results from 3 independent experiments, in technical duplicates, were analyzed using the Delta-Delta CT method with 16S as reference gene.

### 4.11. Statistical Analysis

The results are expressed as means ± SD. Data were statistically analysed using a t-test or two-Way ANOVA, as necessary. Statistical analyses and graphing were done using PRISM4 software (GraphPad Software, La Jolla, CA, USA). A *p* value < 0.05 was considered statistically significant.

## Data Availability

The data presented in this study are available on request from the corresponding author.

## Supplementary Materials

Supplementary materials can be found at https://www.mdpi.com/1422-0067/22/4/1851/s1.
