# Supplementary material for: TFF1 Induces Aggregation and Reduces Motility of Helicobacter pylori
Source: Int J Mol Sci. 2021 Feb 12;22(4):1851. doi: 10.3390/ijms22041851 (PMC7918695; doi:10.3390/ijms22041851)
Supplement: Supplementary file 1 [file ijms-22-01851-s001.pdf]

TFF1 induces aggregation and reduces motility of *Helicobacter pylori*

Daniela Eletto [1#], Megi Vllahu [1#], Fatima Mentucci [1,2], Pasquale Del Gaudio [1], Antonello Petrella [1], Amalia Porta[1,*] and Alessandra Tosco [1,*]

[1] Department of Pharmacy, University of Salerno, Fisciano (SA), Italy
[2] PhD Program in Drug Discovery and Development, University of Salerno, Fisciano (SA), Italy

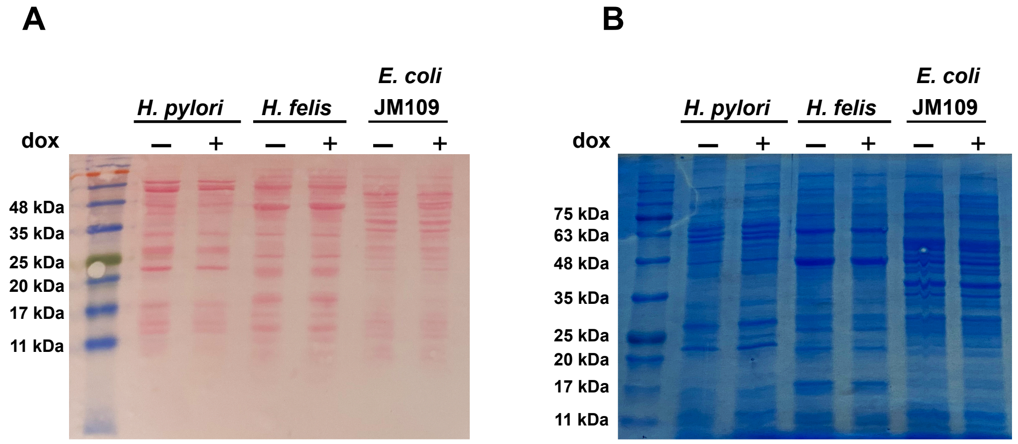

Supplementary Figure 1. Loading control. (A) Ponceau Red staining of the Western blot membrane of Fig. 1A. (B) Coomassie blue staining of the same samples of panel A loaded on a 12 % SDS-PAGE

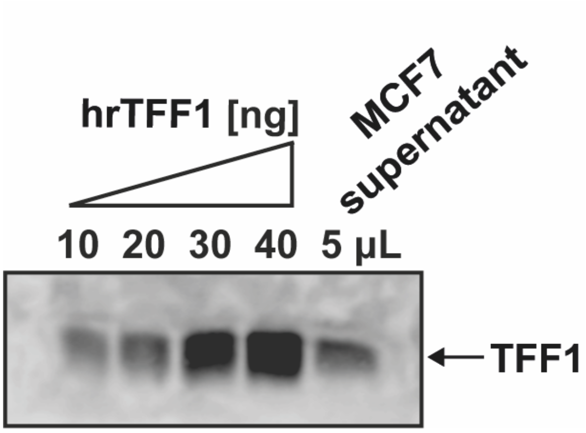

Supplementary Figure 2. TFF1 quantitation in MCF7 supernatant. Western blot analysis of different quantities of hrTFF1 and 5 µL of MCF7 supernatant.

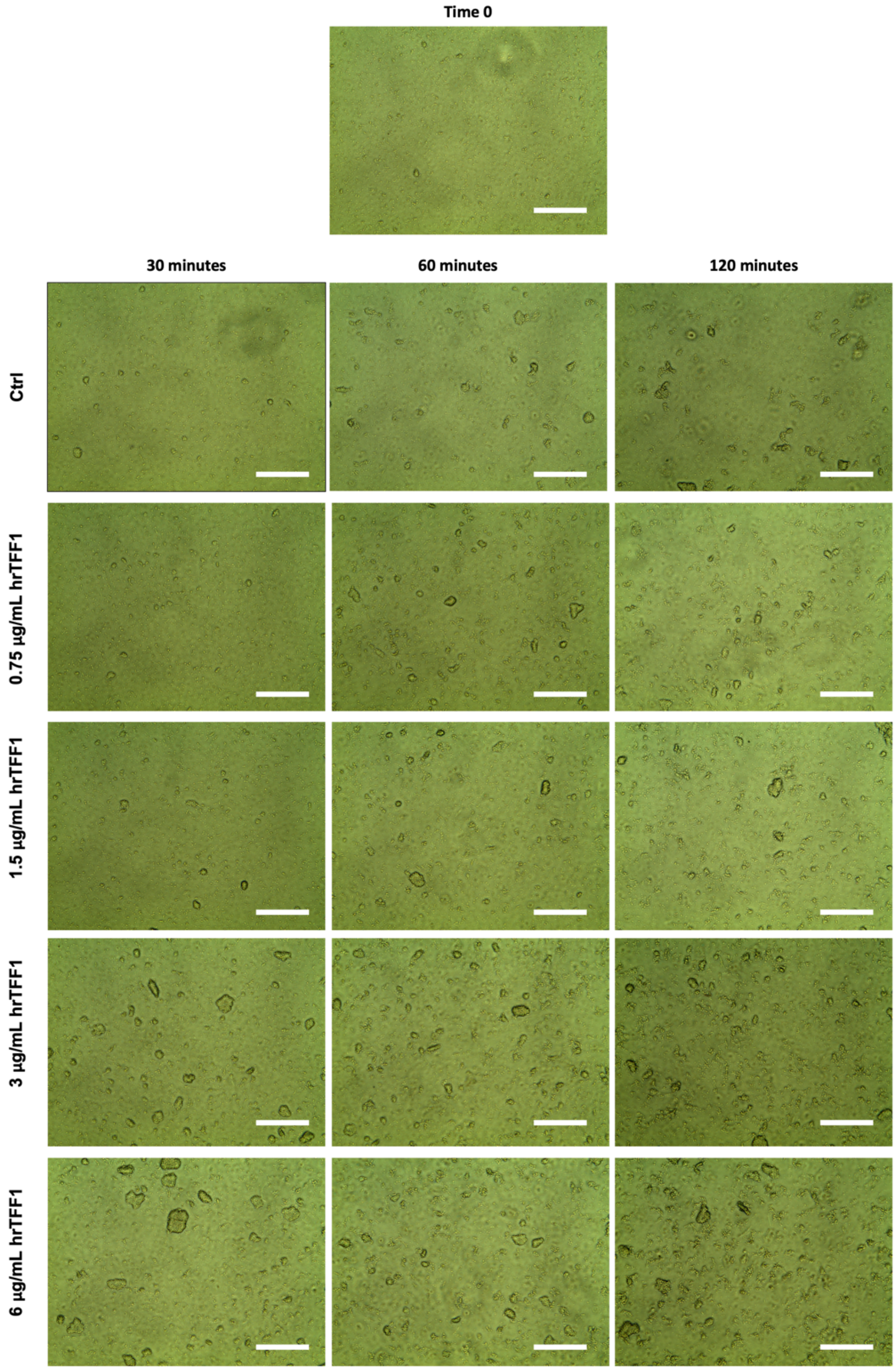

Supplementary Figure 3. hrTFF1 induces formation of *H. pylori* aggregates in time and concentration dependent manner. Optical images of *H. pylori* incubated with different concentrations of hrTFF1 (0, 0.75, 1.5, 3, 6 µg/mL) for different times (0, 30, 60, 120 minutes) . Scale bar 150 µm.

A

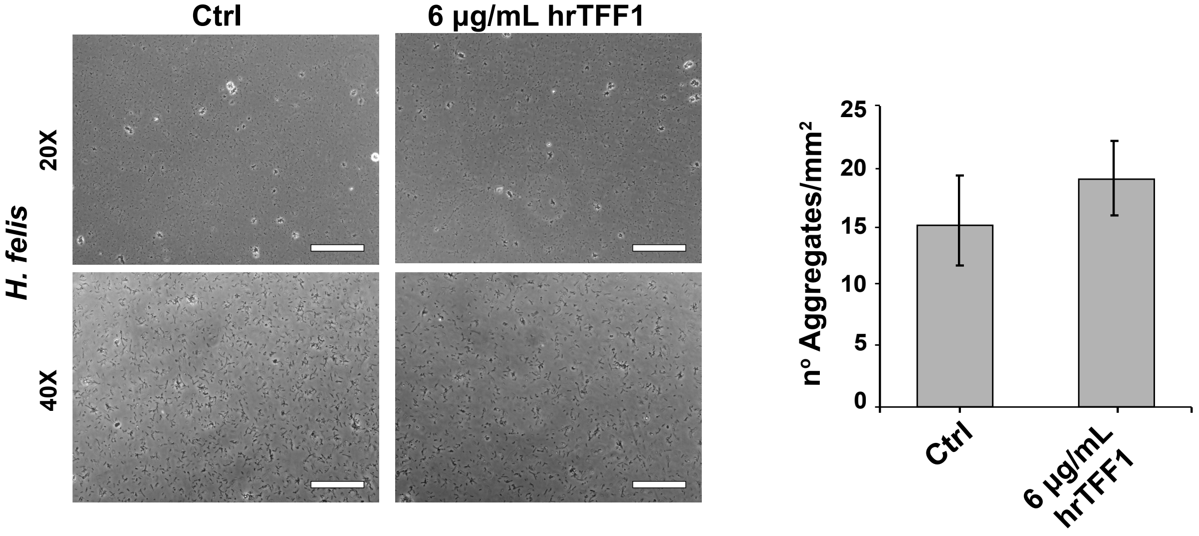

Supplementary Figure 4. hrTFF1 does not induce formation of *H. felis* aggregates. Optical images of *H. felis* incubated with 6 µg/mL of hrTFF1 for 4 h. both at 20X magnification (scale bar 150 µm) and at 40X magnification (scale bar 50 µm). Right panel reports the histograms quantitation of bacterial aggregates.

Gene		Sequence
flaA	Fw	5′-CGGGCAAGCGTTATTGTCTG-3′
	Rv	5′-GCGATACGAACCTGACCGAT-3′
flaB	Fw	5′-ATTCGCAACGCTAATGACGC-3′
	Rv	5′-GGCTTGAACGGCTTTGGTTT-3′
flgE	Fw	5′-GCAGAAAACAGCACGCTTGA-3′
	Rv	5′-ATCGCATTTTTCGCCGCTAC-3′
virB11	Fw	5′-TTAGGCGAAATTGACACGCA-3′
	Rv	5′-ATCATTCCGCTATGCCCAGT-3′
alpA	Fw	5′-ACTACGGCACGAACACCAAT-3′
	Rv	5′-GACCATCTGAACCAGTCGCA-3′
alpB	Fw	5′-CAATAACCAAGCGGGTGGGA-3′
	Rv	5′-TAAAGCGGCGTCCAAAAACG-3′
hopZ	Fw	5′-TGGGGCTGTGGAATGTCATC-3′
	Rv	5′-ATACTCGTGGAATGCGACCC-3′
ureA	Fw	5′-AGACATCACTATCAACGAAGGC-3′
	Rv	5′-TTTCTTCGCCAGGCTCAAAC-3′
vacA	Fw	5′-TGGATAGTGCGACTGGGTTT3′
	Rv	5′-GGCGCTCTTTGAATTGCTCT-3′
cag1	Fw	5′-CGGTGCTATGGGGATTGTTG-3′
	Rv	5′-GCTTCAGTTGGTTCGTTGGTAA-3′
cagA	Fw	5′-AGAGCCTACTGGTGGGGATT-3′
	Rv	5′-AGCCTTGTATGTCGGTGGTG-3′
cag25	Fw	5′-CAAGAATCACTGACAGCTACAAGAA-3′
	Rv	5′-TTTGACCCCTAAAGCGCAAC-3′
16S	Fw	5′-ACGCATAGGTCATGTGCCTC-3′
	Rv	5′-GTGTCCGTTCACCCTCTCAG-3′

Supplementary Table 1. Real-Time PCR primers
